# Risk factors and nursing countermeasures of postoperative pulmonary infection in patients with breast cancer: A retrospective analysis

**DOI:** 10.1097/MD.0000000000026952

**Published:** 2021-09-17

**Authors:** Jinqin Xie, Yanmei Li, Manxia Qiu, Xin Liu, Shuai Zhou, Jinfang Jiang

**Affiliations:** a Department of Oncology, Guangxi Tumor Hospital, China; b Department of Oncology, The First People's Hospital of Yulin, China; c Department of Oncology, The First People's Hospital of Nanning, China; d The Graduate School of Guangxi Medical University, China.

**Keywords:** breast cancer, care, mastectomy, pulmonary infection, treatment

## Abstract

It is necessary to elucidate the potential risk factors of pulmonary infection to provide references for the management of breast cancer.

Our study was a retrospective design, patients who underwent modified radical mastectomy for breast cancer in our department of breast surgery from January 2019 to November 2020 were included. The personal and clinical data of included patients with and without pulmonary infection were compared.

A total of 234 patients with radical mastectomy were included, the incidence of pulmonary infection was 15.38% with 95%confidence interval (CI) 11.42% to 18.98%. There were significant differences in the age, body mass index, diabetes, duration of surgery, combined radiotherapy and chemotherapy, and duration of drainage between patients with and without pulmonary infections (all *P *< .05). Logistic regression analysis indicated that age ≥55 years (odds ratio [OR] 2.128, 95%CI 1.105–3.426), body mass index ≥ 24 kg/m^2^(OR 2.344, 95%CI 1.031–3.299), diabetes (OR 2.835, 95%CI 1.132–4.552), duration of surgery ≥120 minutes (OR 1.394, 95%CI 1.012–1.044), combined radiotherapy and chemotherapy (OR 3.122, 95%CI 1.124–5.273), duration of drainage ≥5 days (OR 1.851, 95%CI 1.112–2.045) might be the independent risk factors of pulmonary infection in patients after radical mastectomy(all *P* < .05). *Pseudomonas aeruginosa* and *Klebsiella pneumoniae* are the most commonly seen bacteria.

The incidence of postoperative pulmonary infections in breast cancer patients is high, and there are many associated risk factors. The perioperative management of patients should be strengthened targeted on those risk factors in clinical practice.

## 1 Introduction

Breast cancer is one of the common malignant tumors in clinical practice. It mostly occurs in women aged 40 to 60, with an incidence rate of 7% to 10%, ranking first among all female malignancies.^[1–3]^ In recent years, the incidence of breast cancer in China has increased at a rate of 3% per year, causing serious harm to women's physical and mental health.^[4]^ The main characteristics of breast cancer are the long course of the disease and poor patient prognosis.^[5]^ Currently, radical mastectomy is one of the main treatments for breast cancer in China.^[6,7]^ However, because radical mastectomy for breast cancer is traumatic and has severe tissue damage, it is easy to cause limited limb function and postoperative pain, which affects the quality of life of the patient.^[8–10]^ Moreover, the resection range of radical mastectomy for breast cancer is large, which will damage the patient's appearance, cause anxiety, worry, and other negative emotions, and affect the prognosis of the patient.^[11]^ There is an association between surgical infection and adverse cancer outcomes in breast cancer patients.^[12]^ Therefore, the prevention and treatment of postoperative complications of breast cancer patients are of great significance to the prognosis of patients.^[13]^

Pulmonary infection is a common postoperative complication in patients with breast cancer, and its incidence is reported to be 11.26% to 20.19%.^[14,15]^ Previous studies^[16–18]^ have shown that complicated pulmonary infection after radical mastectomy seriously affects the prognosis of patients and can even lead to death. Therefore, actively looking for the risk factors of postoperative pulmonary infection to timely and effective prevention and treatment is of great significance for improving the prognosis of patients.^[19,20]^ To this end, we aim to review and analyze the clinical data of patients undergoing radical mastectomy in our hospital, to analyze the risk factors of patients with pulmonary infection after radical mastectomy, and provide evidence support for the clinical treatment of breast cancer.

## 2 Methods

In this study, all methods were performed and reported in accordance with the Strengthening the Reporting of Observational Studies in Epidemiology statement.^[21]^ Our study had been checked and approved by the ethics committee of Guangxi Tumor Hospital (approval number: 1800124. Location: Naning, China. Date: November 28, 2018). Written informed consents had been obtained from all the included patients, we had obtained patient consent to treatment, and we had de-identified all the patient details in this present study.

### 2.1 Patients

Our study was a retrospective study design, we selectively included patients who underwent modified radical mastectomy for breast cancer in our department of breast surgery from January 1, 2019 to November 30, 2020 as the research populations. The inclusion criteria of the patients were as follows: all patients were diagnosed as breast cancer patients by surgery and pathology. All patients were treated with radical mastectomy for breast cancer in our hospital. The personal and clinical data of all patients were complete. The exclusion criteria of this study were: patients with severe acute infection, patients with lung diseases such as bronchitis and pneumonia, patients with hematological diseases, patients with other malignancies, patients with incomplete clinical data, and patients who were unwilling to participate in this study. We did not include patients undergoing immediate implant-based breast reconstruction in this present study.

According to whether the patient had a pulmonary infection during the postoperative hospitalization, the patients were divided into the infection group and the no-infection group. The pulmonary infection was diagnosed according to the diagnostic criteria^[22]^ in China: all infections were diagnosed by bacterial culture and laboratory examination, body temperature ≥38°C, white blood cell ≥10.0 × 10^9^/L, X-ray showed lung inflammation change. The follow-up period in which pulmonary infection was identified in our study was January 1, 2019 to November 30, 2020.

### 2.2 Pathogenic bacteria and fungi analyses

All patients with infection symptoms underwent pathogenic bacteria detection in our study. The pathogenic bacteria were detected as follows: After the patient wakes up in the morning, we collected sputum specimens and place them in sterile culture flasks for bacterial culture. The Mingrui automatic microbial identification instrument (Hensheng Biotechnology Co., Ltd, Shanghai, China) was used to identify the bacterial species. The cultivation and identification of pathogenic bacteria were all conducted by professional doctors in the laboratory of our hospital.

The identification principle for pathogenic fungi was conducted in our laboratory. Yeast-like colonies cultivated on the primary culture medium should be classified and purified before identification. After eliminating bacterial and other fungal contamination and identifying mixed infections, pure colonies would be identified. After the primary culture, it can be identified to the level of genus according to the morphological characteristics. Manual fungal identification and automatic fungal identification system (Mbio 1200, Shanghai, China) were used for the fungal identifications.

### 2.3 Data collections

Two authors collected the personal and clinical data of patients, which included: age, body mass index (BMI), cases of alcohol drinking, smoking, hypertension, diabetes, hyperlipidemia, Tumor Node Metastasis (TNM) staging, duration of surgery, estimated blood loss, combined radiotherapy and chemotherapy, and duration of drainage.

### 2.4 Statistical analysis

In this study, SPSS 21.0 software was used to analyze the obtained data. The measurement data were expressed as “mean ± standard deviation,” and the difference between the 2 groups was analyzed by *t* test. The count data were expressed as rate (%), and the comparison of sample rate was tested by the Chi-square method. Variables with significant differences in the univariate analyses were further included in the logistic multivariate regression analysis to analyze the risk factors of pulmonary infection after breast cancer surgery. We have checked for the goodness of fit of the regression model with *R*^2^. The cutoff value corresponding to the maximum Youden index was considered as the best cutoff value. In this study, the differences between groups were statistically significant with *P* < .05.

## 3 Results

### 3.1 The characteristic of included patients

A total of 234 patients with radical mastectomy for breast cancer were included, of whom 36 patients had pulmonary infection after surgery, the incidence of pulmonary infection in patients after radical mastectomy was 15.38% with 95% confidence interval (CI) 11.42% to 18.98%. As presented in Table 1, there were significant differences in the age, BMI, diabetes, duration of surgery, combined radiotherapy and chemotherapy, and duration of drainage between patients with and without pulmonary infections (all *P* < .05), and no significant differences in the alcohol drinking, smoking, hypertension, hyperlipidemia, TNM staging, and estimated blood loss were found between 2 groups (all *P* > .05).

**Table 1:** The characteristics of included patients.

Variables	Infection group (n = 36)	No-infection group (n = 198)	*t*/χ^2^	*P*
Age (yrs)	59.23 ± 4.98	52. 13 ± 6.12	1.551	.012
BMI (kg/m^2^)	25.33 ± 1.19	23.01 ± 1.35	6.046	.031
Alcohol drinking	15 (41.67%)	79 (39.90%)	1.128	.067
Smoking	7 (19.44%)	36 (18.18%)	1.095	.107
Hypertension	19 (52.79%)	82 (41.41%)	1.249	.054
Diabetes	22 (61.11%)	60 (30.30%)	1.184	.025
Hyperlipidemia	13 (36.11%)	64 (32.32%)	1.212	.079
TNM staging				
I	1 (2.78%)	21 (10.61%)	1.492	.086
II	26 (72.22%)	139 (70.20%)		
III	9 (25%)	38 (19.19%)		
Duration of surgery (min)	148.71 ± 32.44	103.21 ± 30.92	12.563	.032
Estimated blood loss (mL)	385.13 ± 44.57	377.08 ± 39.25	30.128	.096
Combined radiotherapy and chemotherapy	35 (97.22%)	95 (47.98%)	1.727	.007
Duration of drainage (d)	7.57 ± 2.44	3.02 ± 1.85	1.063	.014

TNM = Tumor Node Metastasis

### 3.2 Risk factors of pulmonary infection in patients after radical mastectomy

The results of univariate analyses were present in Table 2. Based on the positive results of univariate analyses, we included age, BMI, diabetes, duration of surgery, combined radiotherapy and chemotherapy, and duration of drainage for further logistic regression analysis. Table 3 presented the variable assignment of multivariate logistic regression. As showed in Table 4, the logistic regression model had the goodness of fit with *R*^2^ = 0.511, logistic regression analysis indicated that age ≥55 years (odds ratio [OR] 2.128, 95%CI 1.105–3.426), BMI ≥ 24 kg/m^2^ (OR 2.344, 95%CI 1.031 3.299), diabetes (OR 2.835, 95%CI 1.132 4.552), duration of surgery ≥120 min (OR 1.394, 95%CI 1.012 1.044), combined radiotherapy and chemotherapy (OR 3.122, 95%CI 1.124 5.273), and duration of drainage ≥5 days (OR 1.851, 95%CI 1.112 2.045) were the independent risk factors of pulmonary infection in patients after radical mastectomy (all *P* < .05).

**Table 2:** The results of univariate analysis.

Variables	OR	95%CI	*P*
Age (yrs)	2.044	1.103–2.831	.018
BMI (kg/m^2^)	1.905	1.317–3.021	.041
Alcohol drinking	1.167	0.511–2.362	.101
Smoking	1.047	0.184–1.752	.074
Hypertension	3.134	0.859–5.238	.106
Diabetes	1.399	1.024–1.941	.009
Hyperlipidemia	2.141	0.747–4.066	.085
TNM staging	1.789	0.225–2.131	.113
Duration of surgery (min)	1.923	1.122–2.501	.034
Estimated blood loss (mL)	2.178	0.857–4.124	.106
Combined radiotherapy and chemotherapy	2.292	1.048–4.791	.012
Duration of drainage (d)	1.554	1.126–2.132	.015

TNM = Tumor Node Metastasis

**Table 3:** The variable assignment of multivariate logistic regression.

Factors	Variables	Assignment
Pulmonary infection	*Y*	Yes = 1, no = 2
Age (yrs)	*X*_1_	≥55 = 1, <55 = 2
BMI	*X*_2_	≥24 = 1, <24 = 2
Diabetes	*X*_3_	Yes = 1, no = 2
Duration of surgery (min)	*X*_4_	≥120 = 1, <120 = 2
Combined radiotherapy and chemotherapy	*X*_5_	Yes = 1, no = 2
Duration of drainage (d)	*X*_6_	≥5 = 1, <5 = 2

**Table 4:** Logistic regression analysis on the risk factors of lower respiratory tract infections.

Variables	β	Wald	OR	95%CI	*P*
Age ≥55 yrs	0.148	0.139	2.128	1.105–3.426	.015
BMI ≥24 kg/m^2^	0.129	0.124	2.344	1.031–3.299	.034
Diabetes	0.131	0.159	2.835	1.132–4.552	.025
Duration of surgery ≥120 min	0.172	0.116	1.394	1.012–1.044	.038
Combined radiotherapy and chemotherapy	0.124	0.121	3.122	1.124–5.273	.014
Duration of drainage ≥5 d	0.132	0.125	1.851	1.112–2.045	.012

### 3.3 Pathogen distributions of pulmonary infection

A total of 36 cases of pathogens had been obtained from laboratory culture. As presented in Table 5, *Pseudomonas aeruginosa* and *Klebsiella pneumoniae* are the most commonly seen bacteria in patients with pulmonary infection after a radical mastectomy.

**Table 5:** The pathogen distributions in patients with pulmonary infection.

Pathogens	Cases	Percentage
Gram-positive bacteria	9	25%
*Staphylococcus aureus*	5	13.88%
Streptococcus	2	5.56%
Enterococcus	2	5.56%
Gram-negative bacteria	25	69.44%
*Pseudomonas aeruginosa*	14	38.89%
*Acinetobacter baumannii*	5	13.88%
*Klebsiella pneumoniae*	6	16.67%
Fungus	2	5.56%
*Candida albicans*	2	5.56%
In total	36	100%

## 4 Discussions

Radical breast mastectomy is a kind of thoracic surgery commonly used for the treatment of breast cancer. It has a great impact on the patient's immune function and respiratory system, and postoperative pain can easily cause sputum and secretions to accumulate in the airway, increasing the patient's risk of postoperative pulmonary infection.^[23–25]^ However, there are few reports on the influencing factors of pulmonary infection after breast cancer surgery, and the related risk factors are still unclear. Therefore, in this study, we have analyzed the factors affecting the postoperative pulmonary infection in breast cancer patients, and the results have showed that age ≥55 years, BMI ≥ 24 kg/m^2^, diabetes, duration of surgery ≥120 min, combined radiotherapy and chemotherapy, and duration of drainage ≥5 days may be the independent risk factors of pulmonary infection in patients after radical mastectomy, early preventative measures, and nursing care targeted on those risks are warranted.

The immunity of the body after radical cancer resection is poor. If combined with radiotherapy or chemotherapy drugs, it is easy to cause immunosuppression and reduce the patient's ability to resist bacteria.^[26]^ Besides, it is easy to damage the respiratory cilia removal system and increase the risk of postoperative pulmonary infection.^[27]^ It is suggested that the clinical need to comprehensively evaluate whether combined radiotherapy and chemotherapy, and when to perform radiotherapy and chemotherapy based on the development of the patient's condition.^[28,29]^ Drainage tubes are often indwelled after radical mastectomy, which affects the healing of wounds to a certain extent. In addition, the drainage port is prone to bacterial invasion, which further increases the risk of postoperative pulmonary infection, prompting the clinical need to do comprehensive nursing work.^[30–33]^ Preventive measures with improved body position, retrograde irrigation and negative pressure drainage, combined with antibacterial drugs, etc may be beneficial to reduce the onset of pulmonary infection.^[34,35]^

Patients after radical mastectomy are prone to pulmonary infection. This study shows that the postoperative pulmonary infection rate is 15.38%. This is basically consistent with the results of previous studies.^[36–38]^ Therefore, the pulmonary infection should be paid attention to by clinicians. This study showed that gram-negative bacteria are the main infectious pathogens, among which *Acinetobacter baumanni* is the common one, gram-positive bacteria are the second, and a small number of patients have fungal infections. The reasons for the high rate of pulmonary infection may be related to the following factors: (1) Patients with breast cancer are older, and their cough and sputum ability are weak, resulting in secretions that cannot be discharged in time and enter the lower respiratory tract, which induces pulmonary infection.^[39–41]^ (2) After the operation, the patient is in a state of stress and the body's immunity is low. If diabetes is combined, the patient's immunity is lower and it is easy to induce lung infection.^[42,43]^ (3) Some patients with a history of smoking have impaired cilia movement in the lungs, making it difficult to effectively remove pulmonary secretions in time.^[44]^ (4) Some patients have used broad-spectrum antibacterial drugs for a long time, and the patients have underlying diseases, which are prone to dysbacteriosis.^[45]^ Therefore, after the patient is admitted to the hospital, the necessary examinations should be actively completed, the abnormal physiological indicators should be corrected in time, including the control of blood pressure, blood sugar, arrhythmia, etc, anemia should be appropriately improved, and intensive care unit monitoring may be necessary after the operation,^[46–48]^ so that the postoperative pulmonary infection may be reduced.

Several limitations in this present study must be concerned. Firstly, our study was a retrospective design, many other variates such as lab test results, pre-operative drug administration, etc could not be included for data analysis, pneumonia may be also associated with the virus infection, which should also be analyzed. Prospective designs are needed to include more variables for analyses in the future. Secondly, the sample size was small in this present study, it may be under appropriate statistical power to detect the group differences. Thirdly, our results might be biased due to the high number of postoperative pulmonary infections, most probable more multimorbid patients were selected as these patients more often have a radical mastectomy. Therefore, future studies with rigorous design and larger sample size in different areas and populations are needed to further elucidate the potential risk factors of postoperative pulmonary infection.

## 5 Conclusions

In summary, based on our results, we have found that age ≥55 years, BMI ≥ 24 kg/m^2^, diabetes, duration of surgery ≥120 min, combined radiotherapy and chemotherapy, duration of drainage ≥5 days may be the independent risk factors that affect postoperative pulmonary infection in breast cancer patients. Clinically, the patient's clinical characteristics and related risk factors should be evaluated to estimate the risk of postoperative pulmonary infection, thereby early prevention and treatment should be performed to reduce the incidence of lung infection and improve the prognosis of patients with breast cancer.

## Author contributions

JJ designed research; JX, YL, MQ, XL, SZ conducted research; JX, YL, JJ analyzed data; JX, YL, MQ wrote the first draft of the manuscript; JJ had primary responsibility for final content. All authors read and approved the final manuscript.

**Conceptualization:** Manxia Qiu.

**Data curation:** Jinqin Xie, Yanmei Li, Manxia Qiu, Jinfang Jiang.

**Formal analysis:** Jinqin Xie, Yanmei Li, Shuai Zhou, Jinfang Jiang.

**Investigation:** Yanmei Li, Manxia Qiu, Xin Liu, Shuai Zhou, Jinfang Jiang.

**Methodology:** Jinqin Xie, Xin Liu.

**Project administration:** Jinqin Xie, Shuai Zhou.

**Resources:** Shuai Zhou, Jinfang Jiang.

**Software:** Manxia Qiu, Shuai Zhou.

**Supervision:** Jinqin Xie, Xin Liu, Jinfang Jiang.

**Visualization:** Xin Liu, Jinfang Jiang.

**Writing – original draft:** Xin Liu.
